# Susceptibility of Mouse Brain to MCMV Infection and Neuroinflammation During Ontogeny

**DOI:** 10.3390/pathogens13121108

**Published:** 2024-12-14

**Authors:** Fran Krstanović, Andrea Mihalić, Lucija Šakota, Berislav Lisnić, Stipan Jonjić, Ilija Brizić

**Affiliations:** 1 Center for Proteomics, Faculty of Medicine, University of Rijeka, 51000 Rijeka, Croatia; fran.krstanovic@uniri.hr (F.K.); andrea.mihalic@uniri.hr (A.M.); lucija.sakota@farf.sum.ba (L.Š.); berislav.lisnic@medri.uniri.hr (B.L.); stipan.jonjic@uniri.hr (S.J.); 2 Faculty of Pharmacy, University of Mostar, 88000 Mostar, Bosnia and Herzegovina; 3 Department of Biomedical Sciences, Croatian Academy of Sciences and Arts, 51000 Rijeka, Croatia

**Keywords:** cytomegalovirus, congenital CMV, neuroinflammation

## Abstract

Human cytomegalovirus (HCMV) rarely infects the brain following infection of adult individuals. However, the virus readily infects the brain during congenital HCMV (cHCMV) infection, frequently causing severe neurodevelopmental and neurological sequelae. Interestingly, although the incidence of cHCMV infection is 0.5–1%, the proportion of congenitally infected individuals in which the virus manages to gain access to the brain is unknown. In this study, we used infection of mice with mouse cytomegalovirus (MCMV), the most commonly used experimental system for modeling HCMV disease in humans, to determine the impact of age on the susceptibility of the brain to cytomegalovirus infection and infection-mediated neuroinflammation. We demonstrate that infection of mice during various stages of neonatal development can lead to CMV neuroinvasion and inflammation. In contrast, MCMV infection does not result in MCMV neuroinvasion and neuroinflammation in weanling and adult mice. The obtained results establish a basis for elucidating the mechanisms of CMV neuroinvasion and the deleterious inflammatory response during ontogeny.

## 1. Introduction

Neurotropic viral infections of the central nervous system (CNS) represent a significant threat to human health worldwide. Prenatal and perinatal viral infections of the brain or systemic viral inflammation can affect fetal brain development, leading to cognitive and developmental disorders or an increased risk of developing neuropsychiatric disorders later in life [1].

Human cytomegalovirus (HCMV) is a prevalent herpesvirus that poses a substantial healthcare burden globally [2]. Primary HCMV infection is usually asymptomatic in immunocompetent individuals due to an effective immune response [3]. Similarly to other herpesviruses, following the resolution of primary infection, HCMV establishes lifelong latency in the host, from which it can periodically reactivate [4]. Primary infection or reactivation of the virus can cause severe disease in immunocompromised individuals, such as transplant recipients, intensive care patients, patients with acquired immune deficiency syndrome (AIDS), and fetuses/neonates [5].

Congenital HCMV (cHCMV) infection is the most common congenital viral infection in developed countries, affecting more newborns than trisomy 21 or fetal alcohol syndrome [6]. Infected newborns may develop severe, life-threatening conditions, permanent neurodevelopmental disorders, visual impairments, and hearing loss [7]. Given the scarce diagnostic and therapeutic options for cHCMV infection, a deeper understanding of the underlying pathogenesis is crucial for developing novel interventions [8]. Research on cHCMV remains mainly limited to observational and histopathological studies [9,10]. Another obstacle in cHCMV research is the strict species specificity of HCMV, which impedes direct investigations of HCMV infection in available animal models of CMV disease. Various animal models have been developed to reduce knowledge gaps regarding the pathogenesis of cytomegalovirus infection, with the infection of mice with mouse cytomegalovirus (MCMV) being the most commonly used and well-established experimental system for modeling CMV infection and disease [11]. To model cHCMV infection, newborn mice are injected intraperitoneally (i.p.) with MCMV on the first postnatal day (PND 1) [12,13]. After the initial infection, MCMV spreads to peripheral organs and migrates to the brain. The infectious virus can be isolated from the brains of infected animals starting from ~7 days post-infection (dpi) up to 21 dpi [13,14]. The exact route of MCMV dissemination to the brain and blood–brain barrier (BBB) crossing remains ill defined; however, it was suggested that the virus can enter the CNS in both cell-free or cell-associated form [11]. On the other hand, MCMV can infect adult mouse brains solely through direct intracranial injection, while peripheral (i.p., intravenous (i.v.), or intranasal (i.n.)) infections in immunocompetent adult mice do not lead to brain infections [15,16]. The underlying differences in differential susceptibility to CMV infection of the brain in neonatal versus adult mice remain elusive. While the incidence of cHCMV is 0.5–1%, the prevalence of brain infection is not known. In one study, HCMV genomes were detected in brains of more than 10% of HCMV-positive individuals [17,18], suggesting that HCMV can infect the brain after birth.

In this work, we systematically analyzed the susceptibility of the brain to MCMV infection in mice in different stages of development and adulthood. We found that MCMV reaches the brain of mice only if they are infected within the first two weeks of postnatal life, while infection in weanling and adult mice does not result in MCMV neuroinvasion. These findings correlate not only with the full maturation of the BBB but also with the maturation of the immune system. Our results, therefore, provide a basis for studying the mechanisms of MCMV neuroinvasion during ontogeny.

## 2. Materials and Methods

### 2.1. Mice

Mice were strictly age-matched within experiments and handled in accordance with institutional and national guidelines. Mice of both sexes were included in all experiments. All mice were housed and bred under specific pathogen-free conditions at the animal facility of the Faculty of Medicine, University of Rijeka, where they were maintained at 22 °C in a 12 h light–dark cycle and relative humidity (40–50%). Wild-type C57BL/6J mice (strain #000664) were obtained from the Jackson Laboratory. The Animal Welfare Committee at the University of Rijeka, Faculty of Medicine, and The National Ethics Committee for the Protection of Animals Used for Scientific Purposes (Ministry of Agriculture) approved all animal experiments (UP/I-322-01/23-01/33).

### 2.2. Viruses and Cell Lines

Tissue culture-derived wild-type (WT) MCMV, reconstituted from BAC pSM3fr-MCK-2fl, was used for the infection in mice [19]. Organ homogenates were titrated on mouse embryonic fibroblasts (MEFs) using standard procedures [19]. Prior to infection, animals were weighed. Mice were i.p.-infected with a weight-adjusted dose of MCMV, which was extrapolated by correlating the weight of adult mice and infection dose 2 × 10^5^ PFU and 1-day-old mice infected with 200 PFU [19]. Therefore, mice infected on PND 1 were infected with a dose of 200 PFU, on PND 4 with 10^4^ PFU, on PND 7 with 3 × 10^4^ PFU, on PND 12 with 4 × 10^4^ PFU, on PND 21 with 6 × 10^4^ PFU, and adult mice (PND 120) with 2.5 × 10^5^ of MCMV.

### 2.3. Flow Cytometry

Mice were sacrificed, and brains were collected in RPMI 1640 with 3% FCS and mechanically dissociated. Single-cell suspensions of brains were prepared according to standard protocols [20]. In brief, 30% Percoll (#17089101 Cytivia, Wilmington, DE, USA) and brain homogenate suspension were underlaid with 70% Percoll in PBS and then centrifuged at 700× *g* for 25 min. Cells in the interphase were collected for the further analysis of microglia and T-cell populations. Flow cytometric analyses were performed using the following anti-mouse antibodies: CD45.2 (clone 104) Alexa Fluor 700 # 56-0454-82 (dilution 1:100), anti-mouse CD11b (clone M1/70) PE-Cyanine7 # 25-0112-82 (dilution 1:400), anti-mouse MHC class II (I-A/I-E) (clone M5/114.15.2) PE-eFluor610 # 61-5321-82 (dilution 1:200), anti-mouse MHC cIass I (H-2Db) (clone 28-14-8) FITC # 11-5999-82 (dilution 1:100), anti-mouse MHC cIass I (H-2Kb) (clone AF6-88-5.5.3) APC # 17-5958-82 (dilution 1:100), anti-mouse CD8a (clone 53-6.7) PE-eFluor610 # 61-0081-82 (dilution 1:400), anti-mouse CD69 (clone H1.2F3) FITC # 11-0691-82 (dilution 1:100), and anti-mouse CD103 (clone 2E7) APC # 17-1031-82 (dilutfion 1:200) purchased from ThermoFisher (Waltham, MA, USA). All data were acquired using a FACSAria (BD Biosciences, Franklin Lakes, NJ, USA).

### 2.4. Statistical Analyses

Statistical analyses were performed with Prism 8 (GraphPad Software Inc., La Jolla, CA, USA). The unpaired two-tailed Student test was used to compare differences between the groups. *p*-values smaller than 0.05 were considered to be statistically significant; * *p* < 0.05; ** *p* < 0.01; ***, *p* < 0.001; **** *p* ≤ 0.0001.

## 3. Results

### 3.1. Age-Dependent Infection of Mouse Brains with MCMV

**Figure 1:**
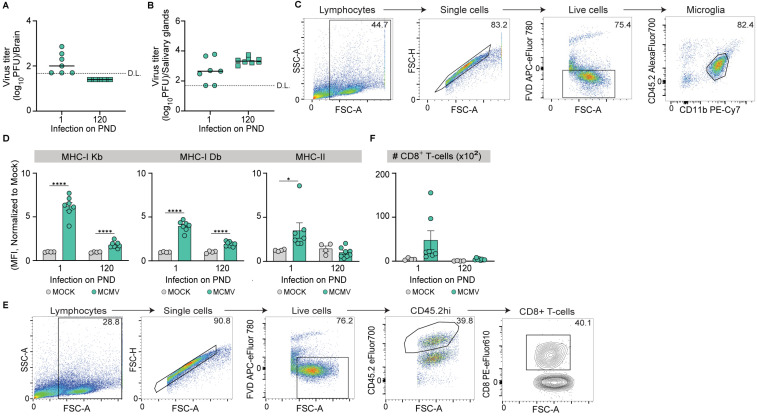
MCMV brain infection and inflammatory responses in newborn and adult mice. Newborn (postnatal day 1 (PND 1)) and adult (PND 120) C57BL/6 mice were infected with MCMV i.p. and sacrificed 10 days post-infection (dpi). Viral titers in (**A**) the brain and (**B**) salivary glands were determined by the plaque assay. Results for individual mice are shown (circles and squares); horizontal bars indicate the median values; D.L., detection limit. (**C**) Gating strategy for microglia (live CD45.2^int^CD11b^+^). (**D**) Normalized expression levels of MHC-I Kb, MHC-I Db, and MHC-II on microglia were determined 10 dpi. (**E**) The gating strategy for CD8^+^ T-cells. (**F**) Absolute numbers of CD8^+^ T-cells were determined at 10 dpi. Mean values ± SEM are shown (*n* = 7–8 mice/time point). Asterisks indicate groups with significant differences in mean MHC-I and MHC-II expression values. * *p* ≤ 0.05, **** *p* ≤ 0.0001.

It is well established that even low doses of MCMV administered i.p. within the first 24 h after birth lead to MCMV infection in the brain [13,14]. In contrast, upon peripheral infection (i.p., i.v., or i.n.) of adult immunocompetent mice, MCMV cannot infect the brain [15,21]. We infected newborn (PND 1) and adult (PND 120) mice to further analyze the susceptibility of brain to MCMV infection. In mice infected on PND 1, we detected the infectious virus in the brain, indicating efficient viral spread and neuroinvasion (Figure 1A). In contrast, the infectious virus was not detected in the brains of adult mice. As expected, infectious virus was detected in salivary glands of both adult and newborn mice (Figure 1B).

Viral infections of the CNS trigger neuroinflammation by activating local immune cells and promoting T-cell infiltration [22]. Microglia serve as the resident macrophages of the CNS and are essential for mounting an effective immune response against neurotropic viral infections [23]. During acute MCMV infection in the brain, microglia acquire a proinflammatory phenotype, marked by the expression of major histocompatibility complex (MHC) class I and II and a substantial change in their transcriptome profile [24]. Since microglia activation serves as an indicator of brain infection, we analyzed MHC expression via flow cytometry 10 days post-infection (dpi) (Figure 1C). MHC-I Kb and Db molecules were strongly upregulated on microglia following the infection of neonatal mice (Figure 1D). Interestingly, the infection of adult mice also resulted in a minor but significant increase in MHC-I expression despite the lack of infectious virus (Figure 1D). On the other hand, MHC-II was upregulated exclusively upon the infection of neonatal mice but not in adult mice (Figure 1D). T-cells, migrating to the brain, play a critical role in controlling MCMV infection in the brain [20]. We performed flow cytometry analysis to determine the CD8^+^ T-cell infiltration into the brain following the i.p. infection of newborn and adult mice (Figure 1E). Again, an increase in the numbers of CD8^+^ T-cells was observed solely in the brains of mice infected on PND1 (Figure 1F), suggesting that viral infection in the brain tissue is needed to drive T-cell infiltration.

### 3.2. MCMV Infection of Mouse Brain During Ontogeny

**Figure 2:**
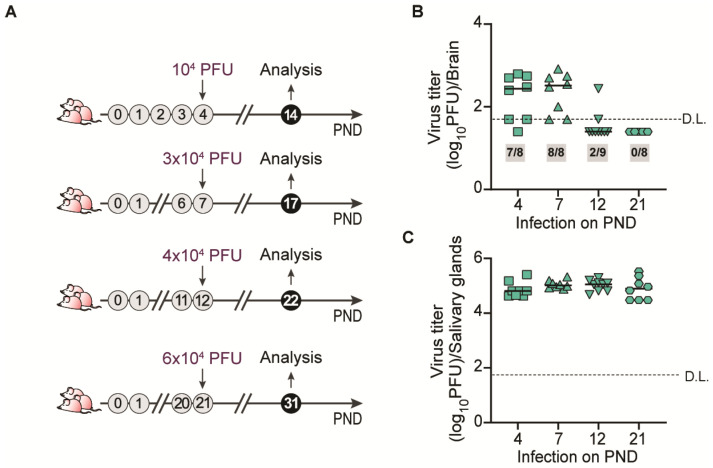
MCMV infection of mouse brain during ontogeny. (**A**) Schematic representation of the experiment. Weight-adjusted doses of MCMV (plaque-forming units, PFUs) used for the infection of mice of different ages are indicated. Viral titers in (**B**) the brain and (**C**) salivary glands 10 days post-infection were determined by the plaque assay (n = 7–9 mice/time point). Numbers in grey boxes indicate the proportion of mice which contained detectable amounts of infection virus in the brain. Results for individual mice are shown; horizontal bars indicate the median values; D.L., detection limit.

To determine the precise age of mice at which MCMV invasion of the brain becomes constrained, we infected mice on PNDs 4, 7, 12, and 21. Animals were weighed and i.p.-infected with a weight-adjusted dose of MCMV. Ten days after infection, brains were collected and viral titers were determined (Figure 2A) [13,14]. Infectious MCMV was detected in the brains of mice infected on PND 4 or PND 7 (Figure 2B). In contrast, while the infectious virus was detected in a few mice, in most of the animals infected on PND 12 infectious virus was below the detection limit in the brain (Figure 2B). In mice infected on PND 21, we did not detect any infectious virus in the brain, suggesting that MCMV brain invasion is constrained between PNDs 12 and 21 (Figure 2B). Infectious virus was present in the salivary glands of all tested animals, demonstrating the successful infection of mice of varying age and viral dissemination (Figure 2C).

### 3.3. MCMV Neuroinvasion Results in the Activation of Microglia

**Figure 3:**
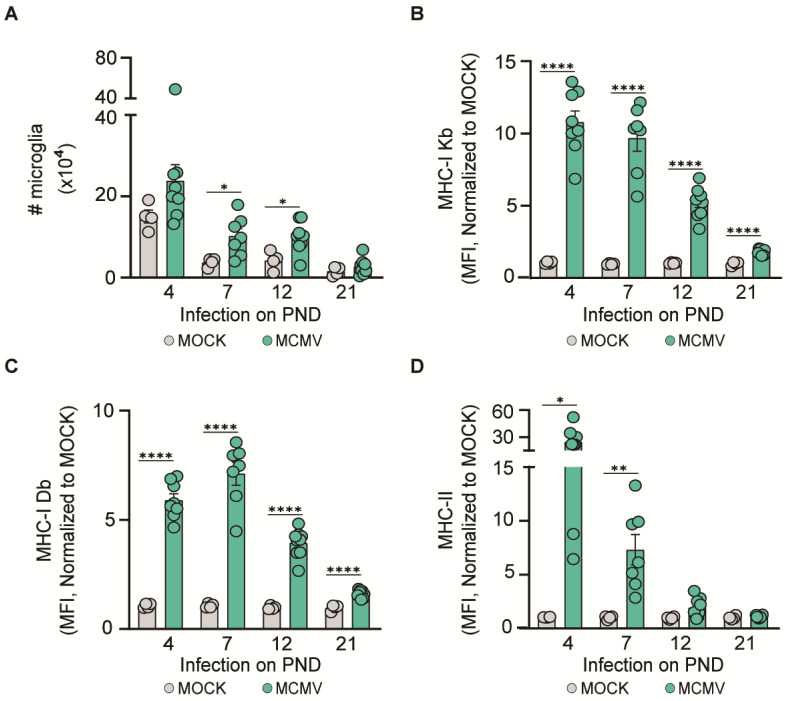
Microglia activation in MCMV-infected brains. C57BL/6 mice were infected with a weight-adjusted dose of MCMV i.p. on a different PND. The number of microglia (**A**) and the expression of (**B**) MHC-I Kb, (**C**) MHC-I Db, and (**D**) MHC-II on microglia were determined at 10 days post-infection. Mean values ± SEM are shown (n = 7–9 mice/time point). Asterisks indicate groups with significant differences in mean MHC-I and MHC-II expression values. * *p* < 0.05; ** *p* < 0.01; **** *p* ≤ 0.0001.

To analyze microglial activation upon MCMV infection during ontogeny, we performed flow cytometry analysis (Figure 3). Increased numbers of microglia were observed in infected mice compared to uninfected animals at all time points except in the group of mice infected on PND 21 (Figure 3A). Similarly, microglia significantly upregulated MHC-I independently of the timing of infection (Figure 3B,C). As observed upon the infection of adult mice, MHC-I was upregulated in mice infected on PND 21, despite the lack of infectious virus in the brain. Finally, MHC-II was upregulated only in infected brains, with no MHC-II upregulation observed in mice infected on PND 21 (Figure 3D). Overall, these data demonstrate that infection of the brain is required for microglial MHC-II upregulation.

### 3.4. MCMV Neuroinvasion Promotes CD8^+^ T-Cell Migration in the CNS

**Figure 4:**
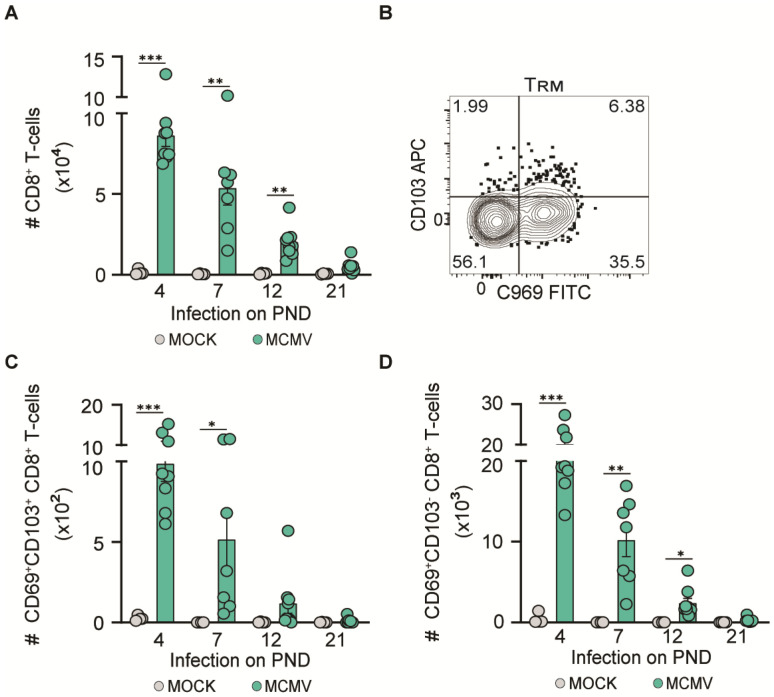
CD8^+^ T-cells migrate to the infected brain. C57BL/6 mice were infected with a weight-adjusted dose of MCMV i.p. at an indicated PND. The number of (**A**) CD8^+^ T-cells, (**C**) CD69^+^CD103^+^ CD8^+^ T-cells, and (**D**) CD69^+^CD103^−^ CD8^+^ T-cells in the brain was determined 10 days post-infection. Mean values ± SEM are shown (*n* = 7–9 mice/time point). (**B**) Representative dot plot of CD103 and CD69 expression using brain CD8^+^ T-cells. Asterisks indicate groups with significant differences in mean MHC-I and MHC-II expression values. * *p* < 0.05; ** *p* < 0.01; *** *p* < 0.001.

To investigate whether MCMV infection during ontogeny promotes CD8^+^ T-cell infiltration into the brain, we performed flow cytometry analysis (Figure 4). The extent of viral infection of the brain (Figure 2B) correlated with the numbers of infiltrating CD8^+^ T-cells (Figure 4A), as well as the acquisition of the tissue resident phenotype (TRM) by CD8^+^ T-cells, (Figure 4B–D). The highest numbers of CD8^+^ T-cells were observed in mice infected on PND 4, followed by a gradual decrease in the mice infected on subsequent PNDs. Importantly, significantly increased numbers of T-cells or TRM cells were not observed in mice infected on PND 21, in accordance with lack of infectious virus in the brain. Thus, MCMV infection in the brain is required for CD8^+^ T-cell recruitment into brain tissue.

## 4. Discussion

HCMV infection during pregnancy can result in brain infections and lead to significant pathologies and severe neurodevelopmental disorders [7,25]. A limited number of studies have reported that HCMV genomes are present in more than 10% of the brains of healthy adults, which is higher than expected based on the prevalence of cHCMV infection [17,18]. However, the mechanisms of neuroinvasion and the extent of postnatal HCMV neuroinvasion remain unclear. To establish when the MCMV infection of the brain is constrained during the postnatal period, we infected mice at different stages of postnatal development (PNDs 1, 4, 7, and 12), juvenile/weanling mice (PND 21), and adult mice (PND 120). MCMV efficiently infects the brains of neonates spanning from early (PND 1) to late (PND 12), but not the brain of weanling mice or adult mice. The underlying causes of differential susceptibility to MCMV brain infection remain elusive. We anticipate inefficient peripheral immune control and/or insufficient BBB integrity could be responsible.

Since CMV is an opportunistic pathogen, neuroinvasion is common in immunocompromised individuals, such as AIDS patients, highlighting the need for effective peripheral immune control [26]. CMV-infected children can shed the virus in bodily fluids up to 5 years following infection, suggesting that virus-specific immune response is inadequate during early childhood [27,28]. It is well established that newborns have an inherently weakened immune response, significantly increasing their susceptibility to viral infections [29]. In addition, it appears that even though the early CD8^+^ T-cell response to MCMV infection is deficient, it still contributes to protecting neonatal mice against the virus [20,30]. Similarly, newborns with cHCMV infection have altered T-cell responses compared to infected adults [31]. Therefore, enhancing early CD8^+^ T-cell responses through vaccination could be beneficial in preventing CMV neuroinvasion [32]. While the exact mechanisms of dampened T-cell responses remain unknown, immunomodulators such as cellular prion protein (PrP) can tamper with neonatal T-cell responses during early CMV infection [33]. Furthermore, the functionality of NK cells is impaired during MCMV infection in newborn mice [34,35]. Finally, the brain in adult mice can be infected with MCMV following systemic or i.n. infection only in the case of severe combined immunodeficiency (SCID) mice, further emphasizing the importance of peripheral immune response for restricting MCMV neuroinvasion [15]. Overall, a developing immune response in the early stages could be insufficient in combatting CMV infections, which may facilitate viral spread to the CNS.

The BBB is a complex, highly dynamic, semipermeable membrane structure that protects the brain from harmful pathogens and molecules. Both in humans and rodents, the BBB develops during gestation; however, it remains leaky during early life [36]. Functional maturation of the mouse BBB occurs after birth between days 12 and 24, while the fetal BBB permeability resembles that of the adult but has increased permeability for small molecules [37,38]. While viruses have developed a broad palette of mechanisms to invade the CNS, inflammation can also increase BBB permeability [39,40]. Free virions can cross the BBB via transcytosis without causing a productive infection [41]. Limited data on adult animals have shown that BBB permeability can be altered with repeated MCMV infections [42]. However, peripheral MCMV infection in adult SCID mice showed no BBB disruption, suggesting that peripheral inflammation could be needed to disrupt BBB integrity [15]. Accordingly, the mechanical or chemical disruption of the BBB integrity can enhance MCMV neuroinvasion [43,44]. Altogether, inflammation or/and BBB immaturity may mediate CMV neuroinvasion.

An ex vivo study has demonstrated that neonatal brain slices are inherently more susceptible to MCMV infection than brain slices of weanling mice [45]. Moreover, weanling mice were found to be more resistant to brain infection by other DNA and RNA viruses than neonates [46,47]. While the underlying mechanisms are largely unknown, interestingly, in the case of the Semliki Forest virus, these age-related differences do not seem to be due to the immune system or permeability of the blood–brain barrier but to the maturity of neuronal cells [46,47]. Thus, differential age-dependent susceptibility to MCMV brain infection could be due to differences unrelated to either the immune system’s maturation status or BBB integrity.

The microglia upregulation of MHC-II and CD8^+^ T-cell infiltration were reported in the brains of cHCMV cases [48,49]. Additionally, CMV-seropositive individuals suffering from psychiatric disorders show increased microglial activation [50]. The presence of MCMV in the brains of animals infected from PND 1 to PND 12 resulted in smicroglial activation, as indicated by strong MHC-I and MHC-II upregulation, along with CD8^+^ T-cell brain infiltration, demonstrating virus-induced inflammation. In contrast, mice infected on PND 21 or PND 120 did not display microglial MHC-II expression or T-cell infiltration in the brain in accordance with the lack of infectious virus. In contrast, a minor increase in the expression of MHC-I molecules was observed, even in adult mice and mice infected on PND 21. During systemic infections, pro-inflammatory cytokines are released into the bloodstream and can cross the BBB, activating microglia [51]. Thus, this minor increase in MHC-I could be due to peripheral cytokines crossing the BBB. In contrast to our study, infection with a low-dose of non-neurotrophic H1N1 strain of influenza A virus (IAV) induced the activation of microglia, which expressed MHC-I and MHC-II, and other markers of activation such as CD80 and F4/80 [52]. Thus, the underlying mechanisms of differential activation of MHC-I and MHC-II upon MCMV infection will be addressed in future studies.

Overall, we demonstrate differences in the MCMV infection of mouse brains at different stages of postnatal development and the associated induction of local immune response and inflammation. These results establish a basis for subsequent studies to elucidate mechanisms of CMV neuroinvasion and the deleterious inflammatory response during ontogeny.

## Data Availability

The original contributions presented in this study are included in the article. Further inquiries can be directed to the corresponding author.
